# Distance and Size Perception in Astronauts during Long-Duration Spaceflight

**DOI:** 10.3390/life3040524

**Published:** 2013-12-13

**Authors:** Gilles Clément, Anna Skinner, Corinna Lathan

**Affiliations:** 1 International Space University, Parc d’Innovation, 1 rue Jean-Dominique Cassini, Illkirch-Graffenstaden F-67400, France; 2 AnthroTronix, Inc., 8737 Colesville Road, Suite L203, Silver Spring, MD 20910, USA; E-Mails: askinner@atinc.com (A.S.); clathan@atinc.com (C.L.)

**Keywords:** human visual perception, gravity, mental representation, distance perception

## Abstract

Exposure to microgravity during spaceflight is known to elicit orientation illusions, errors in sensory localization, postural imbalance, changes in vestibulo-spinal and vestibulo-ocular reflexes, and space motion sickness. The objective of this experiment was to investigate whether an alteration in cognitive visual-spatial processing, such as the perception of distance and size of objects, is also taking place during prolonged exposure to microgravity. Our results show that astronauts on board the International Space Station exhibit biases in the perception of their environment. Objects’ heights and depths were perceived as taller and shallower, respectively, and distances were generally underestimated in orbit compared to Earth. These changes may occur because the perspective cues for depth are less salient in microgravity or the eye-height scaling of size is different when an observer is not standing on the ground. This finding has operational implications for human space exploration missions.

## 1. Introduction

Exposure to microgravity during spaceflight is known to elicit orientation illusions, errors in sensory localization, postural imbalance, changes in vestibulo-spinal and vestibulo-ocular reflexes, and space motion sickness [1]. Recent experiments have also shown that cognitive processes such as face recognition [2], mental rotation of two- or three-dimensional (3D) objects [3,4,5], and judgments of direction and orientation [6,7] are affected during spaceflight.

The 3D world in which we normally act is an elaborate perceptual representation using a product of sensory and neural processes that have been perfected by millions of years of evolution. The study of mental representation of space is concerned with how people make judgments about size and distance, as well as the global shape and scale of visual space [8].

In previous studies we have shown that the occurrence of geometric illusions based on perspective was less frequent in vestibular patients who presented central signs of otolith disorders [9], in healthy observers tilted relative to gravity on Earth [10], as well as in astronauts on board the International Space Station (ISS) [7]. In an earlier report on two astronauts, we observed that when drawing a Necker’s cube, *i.e.*, a 2D image perceived as a 3D object, the height of the drawing was 9% shorter than its width in zero gravity (0G) [11]. This result suggests that an alteration in the mental representation of space is taking place during exposure to microgravity.

The objective of this study was to further test the extent of alterations in the perception of objects and to distinguish motor *vs*. perceptual-motor and cognitive effects. Experiments on board the ISS were performed with the astronauts free-floating and in darkness, which eliminated static otolith, somatosensory, and visual orientation cues. The astronauts were asked to evaluate the size of cubes of various dimensions, or to draw a perfect cube on a digitizing tablet. This protocol allowed us to compare between cognitive (size perception) and sensorimotor (hand drawing) responses. Because size perception is also related to distance, the astronauts’ abilities to evaluate egocentric distance were compared on Earth and in orbit.

## 2. Background

### 2.1. Distance Perception

Distance perception is the ability for estimating distances between objects in any and all directions relative to an observer’s eye. Absolute distance is the exact distance (e.g*.*, in feet or meters) between the observer’s eye and an object, or between two observed objects. Previous research has demonstrated that horizontal distances are accurately estimated up to 4 m, and underestimated by approximately 10% as distance increases [12,13,14,15]. By contrast, it has been demonstrated that vertical distances are overestimated, by about 30%, especially when looking down [16].

Depth is the distance straight ahead of the observer’s eye, in the direction of or into an object or surface. By definition, depth is looking directly into a hole or tube and estimating forward distances. In this paper, however, the term “depth” will refer to the backward dimension of an object (*i.e.*, the distance from the closest edge of an observed object to the furthest edge of the object within the plane of an observer’s forward gaze), and the term “distance perception” will refer to the judgment of forward distances (*i.e.*, the distance from the observer’s eye to the closest edge of the object).

Accurately evaluating distances requires binocular stereoscopic vision (stereopsis) and, for long distances, other cues. These distance cues include: (a) proprioceptive cues from lens accommodation muscles or eye convergence muscles; (b) disparity of object size on the right and left retina; (c) angular variations—or parallax—when moving the head; (d) texture, luminosity, color, and shading variations of the visual scene; and (e) perspective. Perspective modifies angles, makes parallel lines converge, and compresses grids. Consequently, perspective is at the origin of geometric illusions related to orientations, alignments, and angles of straight lines [17]. Also, because of the perspective effect, depth is generally underestimated. For example, pyramids and mountains look steeper from a distance than when close, and objects look compressed when seen in a telephoto lens that brings the image nearer [18].

### 2.2. Size Perception

Estimates of the size of an object placed at a particular distance can sometimes serve as indirect measures of the apparent distance to the object. The “size-constancy” hypothesis states that people perceive the size of an object by relating its retinal size to its distance [19,20]. This hypothesis predicts that inaccuracies in distance perception will result from inaccuracies in size perception. There is some evidence that distance and size perception are related [21]. For example, observers generally overestimate the size of farther objects. This relation is not linear, though: the size estimates increase by a factor of 3 to 4 as the distance to the object increases by a factor of 10 [22]. Nevertheless, the rule is that when we overestimate the distance of an object, we tend to attribute a larger size to this object (and inversely, when we underestimate the distance of an object, we tend to attribute a smaller size to this object).

However, size perception does not always relate to distance estimates. Geometrical inconsistencies have been found in studies examining the size-constancy hypothesis. For example, when seen from the top of a building, people and vehicles at ground level look smaller than expected. According to the size-constancy hypothesis, since vertical distances are overestimated (see Section 2.1), the people/vehicles on the ground should also look larger than normal. Some authors have proposed that the distance scaling of size is not fully operational when looking up or down because the observer is not viewing the area on the ground around his/her feet and this eye height scaling for distance is missing [16].

Distance and size perception are skills learned through repetitive practice. Normally sighted, binocular and even totally monocular people develop and use effective distance and size perception skills. In microgravity, the environment is not structured with a gravitational reference and a visual horizon, so linear perspective is less relevant. Also, the distance between the eyes and the floor varies when astronauts are free floating; therefore they can not use the eye height scaling to estimate distance and size as on Earth.

### 2.3. Drawing from Memory

Ground-based studies have shown that there is a constancy between perception of 3D objects or scenes and representational drawing of them [23]. If we are trying to draw an object or scene from memory, it is likely that we will draw it on the basis of how we perceived it. That is, we should find in the hand drawing from memory the same size distortions found in the visual tests.

## 3. Material and Methods

### 3.1. Study Participants

Eight astronauts (one woman, seven men) ranging from 45 to 56 years (*M* = 49.4, *SD* = 3.9) were tested before, during, and after a long-duration mission on board the ISS. Mission durations ranged from 57 to 195 days (*M* = 154.4, *SD* = 43.3). Subjects were all tested three times pre-flight (at approximately L-90, L-60, and L-30 days), four times in-flight (FD) and three times post-flight (at R+0 or R+1, R+4, and R+8 days). Additionally, a control population of 91 participants (34 women, 57 men) was tested in normal gravity (1G) on the ground. The average age of participants was 43.2 years (*SD* = 10.9).

Informed consent was obtained from all participants. Study approvals were obtained from the investigators’ institutional review boards, as well as from ESA, NASA, and JAXA medical boards. All participants had normal or corrected-to-normal vision with no known visual or vestibular deficits.

The present experiment was designed to answer the questions of whether distance and size perception of objects were affected in astronauts during long-duration exposure to microgravity. Four tests were performed: cube size perception, cube hand drawing, distance perception with cubes, and distance perception with natural scenes. During all the tests, subjects were wearing a head-mounted display subtending a viewing angle of 30° (Z800 3DVisor, eMagin Corporation, Bellevue, WA, USA). All external visual references were blocked by a fabric cover placed over the head-mounted display. The tests were delivered through custom-made software on a laptop computer. The subjects interacted with the computer by the means of a finger trackball (3G GreenGlobe Co., Ltd., Taiwan).

### 3.2. Cube Size Perception

In the first test, subjects were presented with a stereoscopic view of a cube seen in perspective. The cube was made of white lines on a black background. It subtended a viewing angle of 20 deg at a perceived distance of approximately 50 cm. One dimension of the cube (*i.e.*, its width, its height, or its depth) was clearly shorter or longer than the other two dimensions. The subjects were asked to adjust this dimension of the cube so that it had the same apparent size as the other two dimensions.

During each session, 12 trials were performed, four trials with the width, four with the height, and four with the depth, in random order. For each trial, the size differential was calculated between the final (adjusted) dimension and the reference (normal) dimension of the cube. The responses for all trials were averaged individually and the mean and standard deviation were calculated for each test session. Because the in-flight sessions were not performed on the exact same days from launch for each subject (depending on mission duration and other on-board operations), the responses were binned by flight day (FD) periods, *i.e.*, FD 6–30, FD 38–60, FD 65–120, and FD 133–192.

### 3.3. Cube Hand Drawing

In the second test, a 5-second video clip showing a line-by-line sequential drawing of a Necker cube was displayed in the head-mounted display. The test subjects then drew the same cube in a smooth motion using an electronic pen on a digital writing tablet (Intuos A4, Wacom Co., Ltd., Vancouver, WA, USA) without visual feedback (*i.e.*, a blank screen was displayed within the head-mounted display). The size of the tablet’s active area was 305 × 231 mm and the spatial resolution was 5080 lines per inch. The tablet was attached to the subjects’ thighs by knee-straps. Each cube was drawn six times. There was no time limit imposed for the duration of the drawings. Before the first data collection, subjects practiced by drawing approximately 30 Necker cubes using the procedure described in [11].

In total, 144 cubes were drawn pre-flight (6 trials × 3 sessions × 8 subjects), 192 in-flight (6 trials × 4 sessions × 8 subjects) and 192 post-flight (6 trials × 4 sessions × 8 subjects). For each pre-flight, in-flight, and post-flight cube, the length of the drawn horizontal, vertical, and diagonal lines was measured.

### 3.4. Distance Perception with Cubes

In the third test, subjects were presented with a stereoscopic view of three cubes in perspective. Two cubes each subtended a viewing angle of 5° at a perceived forward distance of approximately 30 cm; the third subtended a viewing angle of 2° at a perceived distance of approximately 60 cm. The distance (and corresponding size) of the far cube, or the distance between the two near cubes, could be adjusted using the finger trackball. The subjects were asked to adjust the position of one cube along the horizontal frontal or sagittal axis so that the apparent distance between all three cubes was equal.

During each session, 12 trials were performed: six along the transversal axis, and six along the depth axis. For each trial the size differential was calculated for the adjusted distance and the true distance between the cubes.

### 3.5. Distance Perception with Natural Scenes

In the fourth test, subjects were presented with 12 stereoscopic (anaglyphs) photographs of natural scenes. The scenes were outdoor photographs of cities, forests, mountains, bridges, towers, *etc*. Small yellow targets were superimposed on easily recognizable landmarks within each scene, e.g., a remarkable building, the end of a bridge, the top of a mountain, or the bottom of a tower. The subjects were asked to estimate the absolute distance between themselves and the target (egocentric distance) using a conventional metric of their choice (e.g., feet, yards, or meters).

Since the photographs were downloaded from the Internet, it was not possible to exactly know the true distances from the landmarks. Therefore, for each of the 12 photographs we calculated the differences between the estimated distances during the pre-flight sessions and those reported during the in-flight or post-flight sessions.

### 3.6. Data Analysis

For each of the four tests above, the responses for all trials were averaged individually and the mean and standard deviation were calculated for each test session. Except for the task of distance perception with natural scenes, the responses were binned by flight day periods, *i.e.*, FD 6–30, FD 38–60, FD 65–120, and FD 133–192. A nonparametric one-way ANOVA was performed using Wilcoxon score. Even if most of the data were distributed normally, nonparametric tests were preferred over parametric tests due to the relatively small number of test subjects. The limit for statistical significance was set at 0.05.

## 4. Results

### 4.1. Cube Size Perception

**Figure 1:**
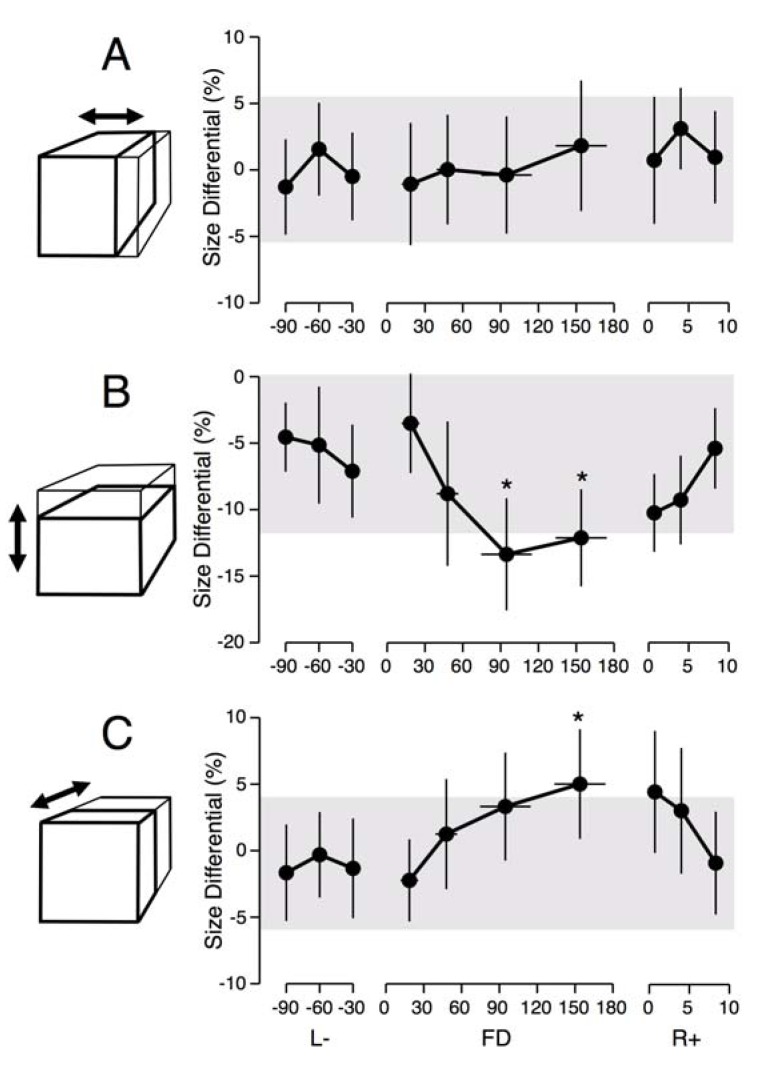
Differences between the width (**A**); height (**B**); and the depth (**C**) of a perfect cube and the cube adjusted by the test subjects. Mean ± SD of four trials for the eight astronauts before (L-), during (FD), and after (R+) a space mission. The shaded area represents the mean ± SD responses for the 91 ground-based controls. * *p* < 0.05 relative to L-30 days.

When comparing the dimensions of the cube that the subjects had adjusted so that it looked normal to them, we found no significant difference between the data collected with the astronauts at L-90 days and with the 91 control participants (Figure 1). In addition there was no difference between the data collected with the astronauts across pre-flight sessions at L-90, L-60, and L-30 days. There was a clear trend for the height of the cube to be smaller and its depth to be larger in-flight compared to pre-flight. A Wilcoxon signed-ranks test for paired differences indicated that the difference in height between the flight day period FD65-120 and the final pre-flight data collection session (L-30) was significantly different from zero (*Z* = 4.24, *p* < 0.001, *r* = 1.5). A significant difference was also observed between the period FD133-192 and L-30 (*Z* = 3.12, *p* < 0.001, *r* = 1.1). The difference in depth between the period FD133-192 and L-30 was also significantly different from zero (*Z* = 3.92, *p* < 0.001, *r* = 1.39).

### 4.2. Cube Hand Drawing

**Figure 2:**
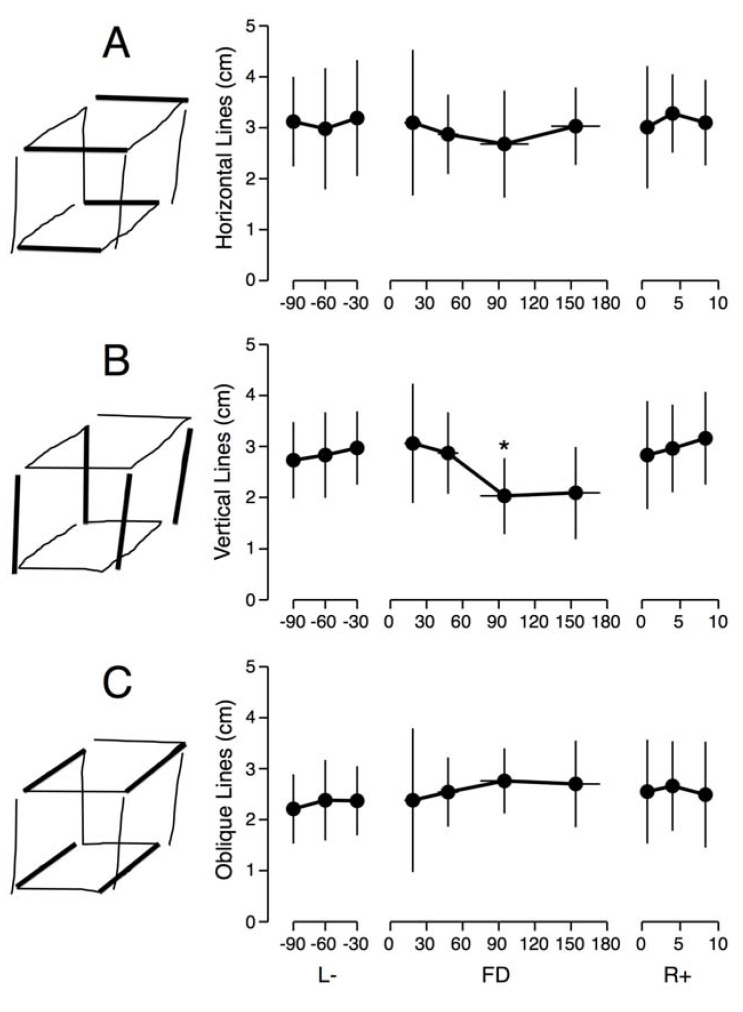
Comparison between the lengths of the horizontal (**A**); vertical (**B**); and oblique (**C**) lines of hand-drawn Necker cubes with the eyes closed. Mean ± SD of six trials for the eight astronauts before (L-), during (FD), and after (R+) a space mission. * *p* < 0.05 relative to L-30 days.

There was no difference in the length of the horizontal, vertical, or oblique lines of the Necker cube drawings between the pre-flight L-90, L-60 and L-30 sessions. During the flight, the data is consistent with the cube size perception data although the effects are not as large. A Wilcoxon signed-ranks test for paired differences indicated that the length of the vertical lines was significantly smaller during the periods FD65-120 (*Z* = 1.69, *p* < 0.04, *r* = 0.6) relative to L-30 (Figure 2). All other paired differences were not significant.

### 4.3. Distance Perception with Cubes

**Figure 3:**
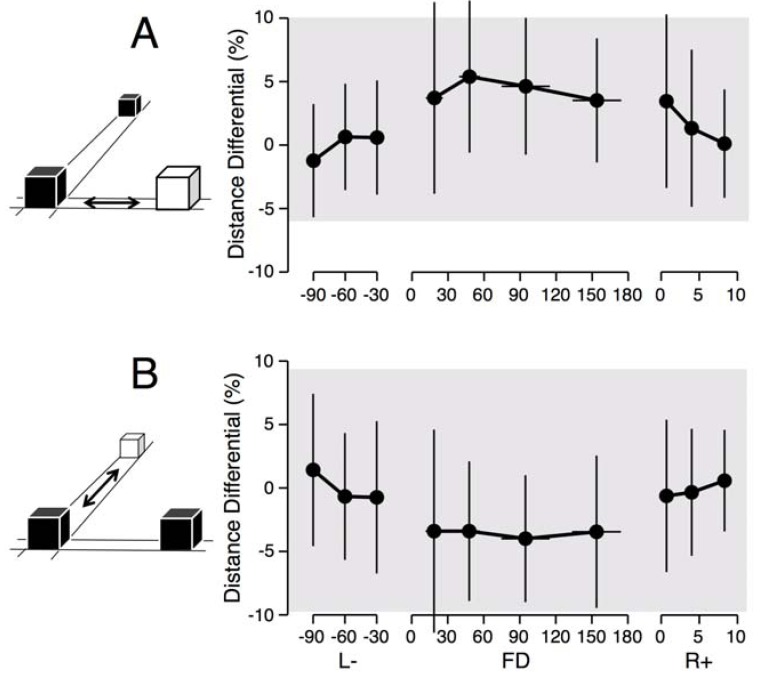
The subjects were instructed to displace the white cube horizontally in the frontal (**A**) or sagittal (**B**) plane to match the distance between the two black cubes. The distance between the two black cubes changed at each trial. Graphs show the differences between the adjusted distance and the true distance. Mean ± SD of six trials for the eight astronauts before (L-), during (FD), and after (R+) a space mission. The shaded area represents the mean ± SD responses for the 91 ground-based controls.

In this test, subjects were asked to estimate the perceived distance of a cube by replicating the extent they were viewing in another direction through visual matching. The true distances ranged between approximately 30 and 60 cm. The perceived distances were fairly accurate on Earth in both the astronauts and the control participants (Figure 3). In orbit, the astronauts underestimated the distance, as consistently shown when adjusting the distance of the cube either along the horizontal frontal or sagittal direction. However, this underestimation was not found to be significant, presumably due to the larger variance in the data for this test, for both the ground-based controls and the astronaut sample.

### 4.4. Distance Perception with Natural Scenes

**Figure 4:**
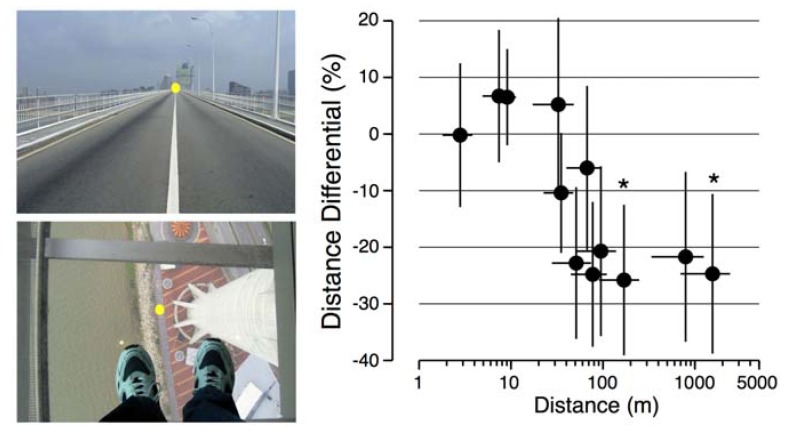
Mean ± SEM of the difference between the perceived distances of targets superimposed on natural scenes before the flight (3 sessions) and during the flight (4 sessions) for the eight astronauts tested. * *p* < 0.05 relative to L-30 days.

Because of the time constraints, only 12 photographs were used: six for testing horizontal distance and six for testing vertical distances. Prior to the flight, the egocentric distances reported by the astronauts ranged from 2 m to 2000 m, with a uniform distribution. All pre-flight measures were not significantly different from each other or from post-flight measures. In addition, these distance estimates were not significantly different from those reported by the 91 participants in the control group. On average, the astronauts reported distances above 50 m to be about 20%–25% smaller in-flight than pre-flight (Figure 4). Wilcoxon tests indicated that this underestimation was significant for distances that were reported to be at 180 m (*Z* = 1.74, *p* < 0.04, *r* = 0.62) and 1500 m (*Z* = 1.82, *p* < 0.03, *r* = 0.64).

## 5. Discussion

We found that the judgment of the size and distance of objects is altered in astronauts following several months in orbit. Visual perception of the height and the depth of objects, as well as the distances of objects are particularly affected. Although our sample of astronaut-subjects is small and only a few differences between pre-flight and in-flight measurements are significant at *p* < 0.05 level, there is a consistent trend in our results that warrants reporting. Our findings suggest that the mental representation of the three-dimensional world changes during spaceflight. This change in scale and shape of astronauts’ visual space may have implications for future human exploration missions.

When astronauts adjusted the size of a cube so that it looked normal in orbit, they made its height shorter and its depth longer than on Earth, which means that a perfect cube was perceived as taller and shallower. We also found the same features in the hand drawing of cubes: the height of hand drawn cubes was shorter in-flight compared to pre-flight. These results indicate that both cognitive and perceptual-motor changes take place during adaptation to spaceflight. The two distance perception tests also indicate that astronauts tend to underestimate the distances of objects located either at very close range (<60 cm) or at long-range (180 m and 1500 m) compared to Earth measurements. Given previous findings that in a terrestrial environment large distances are underestimated by about 10% [15], our results suggest that in orbit the underestimation of true distances in orbit could be as high as 35%.

The increase in perceived height of the cube and the underestimation of its distance are inconsistent with the visual size constancy rule, which predicts that we tend to attribute a smaller size to an object when we underestimate its distance. The perceived depth of the cube, however, decreases in orbit. According to the size-constancy rule, if a 2D object keeps the same aspect (it varies in a homothetic manner) when it moves closer or farther away, a 3D object does not. The 3D shape changes with distance according to perspective. The frontal elements decrease as an inverse function of distance, whereas the depth decreases as an inverse function of the square of the distance. Accordingly, perceived depth should be less affected by distance than perceived height. Indeed, in the hand drawn cubes, a significant effect on depth was not observed.

Another interpretation is related to the observation that, on Earth, the body posture is used to scale the visual space. For example, Linkenauger *et al*. [24] have shown that the perceived distance and size of an object are related to the arm length and the hand size of the participant. Participants who had longer arms perceived targets as closer and the space within which they could reach objects as larger. Additionally, many studies have found that observers can scale the sizes or heights of targets to their eye height [25,26]. Participants use eye height information when standing on the ground plane to scale the distance and size of objects that are also on the ground plane. In our study the astronauts were free-floating, having no contact with the floor, so they could not use the eye height information to scale size or distance.

Distortions of perceived size and shape of objects have previously been reported during spaceflight. For example, the famous inverted-T illusion was less present in astronauts in 0G than in 1G [7], and the latency for detecting a symmetry axis in geometrical figures or recognizing inverted faces was reported to be faster in orbit than on Earth [2]. A decrease in the vertical size of a 3D object drawn in orbit has previously been observed in two astronauts during a 7-day space mission [11]. An underestimation of object distance and size has also been previously measured during short-term exposure to microgravity in parabolic flight [27].

Ground-based studies have long shown that the perceived shape of objects changes when the observers are tilted relative to gravity [20,28]. For example, a square figure in retinal coordinates is perceived as a diamond, or a *d* becomes a *p* with the head tilted by 45° in roll. Recent studies have shown that these effects disappear in microgravity [5,29], which indicate that the gravitational frame of reference on Earth is fundamental for object recognition and identification. Furthermore, Ching *et al*. [30] found an overestimation of distance in prone observers as compared to standing observers.

Numerous studies have also concluded that innate processes of stimulus organization and form description, with the help of past experience, enable humans to reconstruct a perceptual world that adequately corresponds to the objects in the real world [17,23,28]. The results of the “shape from shading” experiment have shown that in orbit astronauts no longer assume that the light shines from “above” (in relation to retinal coordinates) for the image to pop-out [6]. All these studies confirm that gravity plays a major role in visual perception.

Although space research has not demonstrated consistent deficits in 3D perception [1] many astronauts have reported that they tend to underestimate the distance and size of objects. This was particularly evident on the lunar surface [31] as often reportedly occurs on Earth in the clear air and desert landscapes.

During an elegant experiment performed during the Neurolab space mission, crewmembers were required to catch a ball that fell with a constant velocity in 0G (compared to a constant acceleration on Earth). Results showed that they missed the ball by moving their arms too early [32]. The authors surmised that the subjects were using an internal model of gravity and reacted as if the ball was still accelerating downwards. However, the astronauts’ responses could also be due to an underestimation of the ball distance. Similarly, Paloski *et al*. [33] reported that during landings of the Space Shuttle, especially after missions lasting more than 10 days, the vehicle’s vertical speed during the final approach was much faster than during shorter missions and during training simulations. This pattern could also be due to an underestimation of distance, in this case vertical distance between the orbiter and the runway, by the pilots after two weeks in orbit.

The number of subjects and observations within the current study is relatively small, requiring that these findings be confirmed by further investigations. Nevertheless, the fact that the responses of the astronauts on Earth are not different from those of the 91 control participants indicates that this sample is representative of the normal population. Also, the absence of significant differences in the astronauts’ responses to the tests across the L-90, L-60 and L-30 pre-flight sessions and the fact that the post-flight data return to baseline rule out a possible training effect to the repetition of tests.

Another limitation of this study is that the test for distance estimates within natural scenes involved both horizontal and vertical distances. Due to time constraints the number of photographs was limited, eliminating the ability to compare the difference between horizontal and vertical estimates for the same true distance. On Earth, large horizontal distances are typically underestimated by 10%, whereas vertical distances (heights) are overestimated. In addition, vertical distances are overestimated slightly when viewed from the bottom and significantly more so (up to 30%) when viewed from the top [16]. One hypothesis is that when viewing from top or bottom, the horizon and the observer’s eye height are useless cues for distance because the area around his/her feet on the ground plane is not in view. An experiment called Passages is currently studying the eye height scaling effect in astronauts on board the ISS when they attempt to walk through a virtual doorway. If the misperception of horizontal distance in our experiment is indeed due to the absence of an eye height scaling in free-floating astronauts, then the perception of vertical distance should be less affected by spaceflight than horizontal distance. Another interesting question is whether the asymmetry (viewing from the top *vs*. viewing from the bottom) would still be present in microgravity.

Yet another criticism is that the possibility that the effects seen in our study are not due to microgravity but to confinement. Indeed, persons confined to closed modules have a view that is restricted to only a few meters, and the surrounding objects cannot be perceived relative to the horizon. Thus, one could expect changes in visual space perception as a consequence of the prolonged confinement in spacecraft. However, the absence of changes in 3D perception in the participants of a 520-day isolation study simulating a mission to Mars [34] seems to rule out this interpretation.

The current theory of visual perception is that the perceived shape and location of objects is not so much the direct result of certain identifiable stimulus cues as it is a mental construction. For example, cast shadows or objects such as trees and roads suggest a plane on which objects are resting. The plane is thus constructed to encompass these objects. Cues such as size and linear perspective enhance the construction or make it more vivid [23]. Perception is making a remarkably efficient use of often inadequate, and sometimes ambiguous, information for selecting internally stored hypotheses of the current state of the external world. These hypotheses are predicting what kinds of objects are present, and their sizes and positions in space [17]. A given object may have a variety of sizes and a very large range of possible distances. Therefore, current information must be used for setting the size and distances scales. If, for some reason, the size and distance scales are set incorrectly, one should expect to find related perceptual distortions of size or distance. The results of the present study suggest that the rules of geometrical perspective and eye height scaling are less salient in microgravity, and when inappropriate to true distance they produce errors in judging object size.

## 6. Conclusions—Consequences for Human Space Exploration

Distortions of the visual space and misperception of object size, distance, and shape during space missions represent potentially serious operational consequences. For example, if a crewmember does not accurately gauge the distance of a target, such as a docking port or an approaching vehicle then the speed of this target may be misevaluated, leading to operational errors. In fact, it is believed that a poor sense of closing speed was a contributing cause to the collision between a Progress supply spacecraft with the Mir space station in 1997 [35].

Robot operations in space have been increasing with two large robotic manipulators (Canadarm 2 and Dextre) on the ISS structure and robotic arms or cargo cranes on the Russian, European and Japanese laboratories. Telerobotic operations are likely to increase in importance during planetary exploration human missions. Distortions in the mental representation of objects and their surroundings may influence the ability of astronauts to accurately perform cognitive and sensorimotor tasks such as those involved in robot control. In addition, if a normal cube is perceived as taller in height and shallower in depth in orbit compared to Earth, then astronauts may perceive their workspace in orbit to be taller and shallower. This is an interesting concept for designers of space habitats. Even if the effects seen in our study appear to be only about 5% after 90 days of spaceflight, the misperception of depth may cause delays and stress in case of emergency. Finally, linear perspective—the converging projection of parallel contours receding into the distance—is more prevalent in the constructed environment of modern society than in the more natural environment of the Moon or Mars as they are today. Linear perspective has evolved as an innate cue for depth on Earth, and it is likely that it will be less relevant for on the surface of other planets.

The errors in perceived distance and size of objects provide evidence of distortions within the mental representation of spatial cues in the absence of perceived gravity. While these distortions have implications for human performance in space, other mental representation and construct distortions may be even more profound and warrant further exploration and consideration of countermeasures. These include distortions in both visuo-spatial and perceptual-motor constructs such as tactile perception and proprioception, potentially impacting visuomotor mapping and hand-eye coordination. Finally, the evidence presented here may have further implications regarding the plasticity of more complex mental representations and constructs yet to be addressed by future research.
